# A descriptive study of solitary death in Yokohama City

**DOI:** 10.1186/s12199-019-0766-z

**Published:** 2019-02-14

**Authors:** Yasuhiro Kakiuchi, Ryoko Nagao, Eriko Ochiai, Yu Kakimoto, Motoki Osawa

**Affiliations:** Department of Forensic Medicine, Tokai University School of Medicine, 143 Shimokasuya, Isehara, Kanagawa 259-1193 Japan

**Keywords:** Solitary death, Postmortem interval until finding, Marital status, Vital statistics, Emergency transportation data

## Abstract

**Background:**

The solitary death rate in Japan is expected to continue increasing because of its growing super-aged society and the rapid growth of home care in the country. To accurately determine the actual status of solitary deaths, we used a novel analysis method of combining vital statistics and ambulatory care information in Yokohama City.

**Methods:**

Data of persons who died at home in 2013 were obtained from death certificate notifications. We also obtained the emergency transportation records that matched the cases of these death certificate notifications. Then, we gathered information regarding age, gender, marital status, and cause of death for the matched cases.

**Results:**

There were 1890 “suspected unnatural deaths,” in which most solitary deaths could be included, among all citizens who died at home (*n* = 4847). We were able to match 1503 of these cases with emergency transportation records. These 1503 cases were divided into two groups, “solitary death” (*n* = 349) and “un-solitary death” (*n* = 1154) according to the postmortem interval until finding (PMI-f). Pearson’s *χ*^2^ tests conducted for the two groups revealed that there were significant differences regarding the proportion of persons who were elderly, unmarried, male, and had a hepatic disease and senility. A logistic regression analysis also showed that an increased likelihood of a prolonged PMI-f was associated with males and an unmarried status with hepatic diseases.

**Conclusions:**

Unmarried, male sex, and liver diseases are independent risks for solitary death in Yokohama City.

## Introduction

Currently, Japan faces the serious concerns of rapid aging and a very low birthrate. The proportion of elderly people, aged 65 years or older, in the total population was approximately 27.6% in 2017, but it is expected to increase to approximately 33% by 2030 [1]. Particularly, the number of solitary elderly households receiving little social and family support system is increasing yearly and has become a serious social problem that can even include “solitary death” [2, 3], generally defined as people dying alone and remaining undiscovered for a certain period of time.

Meanwhile, previous research has indicated that approximately 50% of Japanese citizens hope to die at home [4]. For most people, spending the last stage of life in a desired location is an important element for achieving a desirable death [5]. Therefore, in line with such expectations among the public, home care is expected to become rapidly more common in the future [6, 7]. On the other hand, the solitary death rate will simultaneously continue to increase.

However, it has been previously noted that the status of solitary deaths in Japan is difficult to fully understand through vital statistics alone [8]. This is because information regarding “households” is missing from the vital statistics data; thus, it is impossible to trace whether the deceased persons were living alone [8]. On the other hand, even for persons who were not living alone, there are a certain number of solitary deaths due to isolation within their households. Thus, it has been strongly argued that, regardless of whether a person lives alone, there are cases that should be considered as solitary deaths when enough time elapsed between the person’s death and the discovery of death [3, 9].

In this study, we adopted the view that the time elapsed between death and discovery of death is important for understanding solitary deaths. We estimated the length of time from the time of death to the discovery of death by comparing copies of death certificate notifications, which are the source data for vital statistics, and emergency transportation records. We then classified persons who died at home into two groups (solitary and un-solitary death) based on the postmortem interval until the body was found. Subsequently, we aimed to elucidate the risk factors of solitary death.

## Material and methods

### Data sources

In this study, to correctly understand the status of solitary deaths, we used a methodology that differed from the conventional analyses that mainly relied on vital statistics alone. We employed an analytical method to perform comparisons and to match emergency transportation data and death certificate notifications gathered from vital statistics. More specifically, with the cooperation of the Medical Care Bureau of Yokohama City, we applied to the Ministry of Health, Labor and Welfare, for the use of death certificate notifications from vital statistic surveys, and we obtained the data of persons who died at home in 2013 in Yokohama City. With the cooperation of the Yokohama City Fire Bureau, we also obtained emergency transportation records that matched the cases from the death certificate notifications. These data are anonymous and are not linkable to specific persons. Then, we gathered information on age, gender, marital status, and cause of death for the matched cases.

### Statistical analysis

The detailed version of the Vital Statistics Survey Death Forms of Yokohama citizens in 2013 was divided into “examined” and “attended” deaths, based on the presence of a postmortem certificate. In Japan, when causes of death found to be obvious disease or natural causes, the deaths were regarded as attended deaths and the death certificates were prepared by general physicians. On the other hand, when causes of death were not immediately found to be disease or natural causes, the deaths were not regarded as attended death but examined one, and the death certificates were prepared by medical examiners, along with police investigation. All postmortem examinations were performed by medical examiners in Yokohama City in 2013. Cases in which medical examiners had prepared death certificates were regarded as examined deaths, whereas cases in which general physicians had prepared the certificates were regarded as attended deaths (i.e., pure natural death). Then, we further sub-divided examined deaths into “pure unnatural deaths” (i.e., homicide, suicide, accidental deaths [e.g., falling and drowning] and deaths from unknown causes) and “suspected unnatural deaths” (i.e., deaths subjected to postmortem examination because the death was either unattended or because of another reason; these deaths were ultimately determined to have been caused by disease or natural causes).

According to a previous study [8], most solitary deaths can be included as “suspected unnatural deaths.” Therefore, we obtained emergency transportation records that matched the suspected unnatural death cases (1503 of the 1890 cases) from the Yokohama City Fire Bureau. Then, those matched cases were divided into two groups, “solitary death” (*n* = 349) and “un-solitary death,” (*n* = 1154) according to the postmortem interval until finding (PMI-f). The PMI-f of the solitary death cases was 3 days or longer, while it was 2 days or shorter for the un-solitary deaths. This is because the dead body generally begins to decompose when PMI-f is 3 days or longer. In such cases, most of the general physicians in Japan refuse to prepare death certificates and the bodies are sent to the medical examiner’s office. In other words, within 2 days after the death, death cases are generally dealt with by general physicians, as well as normal attended death cases, without any police investigations. In conclusion, in this study, we operationally defined “solitary death” as those cases for which PMI-f was 3 days or longer. Finally, information was gathered for both groups in terms of age, gender, marital status, and cause of death, from the detailed version of the Vital Statistics Survey Death Forms of Yokohama citizens in 2013.

A Pearson’s *χ*^2^ test was conducted between two groups with regard to age, gender, marital status, and cause of death. Then, odds ratios (ORs) were calculated by a logistic regression to estimate the strength of association between the PMI-f and age, gender, marital status, and cause of death. PMI-f was used as binary data (3 days or longer as “long, solitary”; 2 days or shorter as “short, un-solitary”) and set as the criterion variable, whereas age, gender, marital status, and cause of death were designated as the explanatory variables. The variance inflation factor (VIF) was used to check for multicollinearity. The significance level was set at 5% for all tests. SPSS (SPSS Statistics 19; IBM, Tokyo, Japan) was used for the analysis.

## Results

**Fig. 1:**
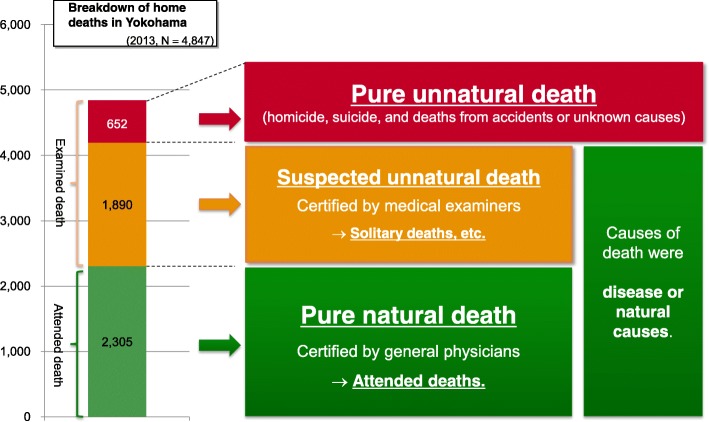
Breakdown of home deaths in Yokohama City in 2013 by type of death

Figure 1 displays the breakdown of the deaths among Yokohama citizens who died at home (*n* = 4847) among all citizens who died in 2013 (*n* = 31,573). There were 2305 attended deaths and 2542 examined deaths. When we further sub-divided examined deaths, we observed 652 “unnatural deaths” and 1890 “suspected unnatural deaths.”

Table 1 summarizes the cases used in this study and illustrates the number of cases, proportion of elderly persons, gender, marital status, and proportion of each cause of death and *p* value (by Pearson *χ*^2^ test) according to the two groups (solitary death and un-solitary death). The results of the Pearson *χ*^2^ test for the proportion of elderly persons, men, and unmarried persons, and autopsies among the two groups revealed that, for a long PMI-f (3 days or longer), the proportion of persons who were elderly (*p* < 0.001) and senile (*p* = 0.001) significantly decreased, while the proportion of unmarried persons (*p* < 0.001), men (*p* < 0.001), and persons with hepatic disease (*p* < 0.001) increased.

**Table 1:** Characteristics of study subjects

Characteristic		Solitary death	Un-solitary death	*p* value
Number of cases		349	1154	
Aging	Aged 65 or older (%)	230 (65.9)	912 (78.9)	< 0.001
Gender	Male (%)	272 (77.9)	691 (59.9)	< 0.001
Marital status	Unmarried (%)	146 (41.8)	193 (16.7)	< 0.001
Cause of death	Cardiovascular disease (%)	196 (56.2)	683 (59.2)	0.315
	Cerebrovascular disease (%)	47 (13.5)	138 (12.0)	0.452
	Hepatic disease (%)	38 (10.9)	45 (3.9)	< 0.001
	Respiratory disease (%)	27 (7.7)	72 (6.2)	0.323
	Malignant neoplasm (%)	18 (5.2)	61 (5.3)	0.925
	Digestive system disease (%)	6 (1.7)	23 (2.0)	0.744
	Senility (%)	2 (0.6)	52 (4.5)	0.001
	Other causes (%)	15 (4.3)	80 (6.9)	0.076

The logistic regression analysis results are shown in Table 2 and illustrate that all of the VIF values were below 10, indicating low multicollinearity. The odds ratios for gender (male vs. female), marital status (unmarried vs. married, divorced, and widowed), and cause of death (hepatic disease vs. all other diseases) were significant (*p* < 0.001, respectively), indicating significant associations with PMI-f. However, there were no significant associations for age (youth vs. elderly, *p* = 0.585).

**Table 2:** Factors associated with postmortem interval until finding (PMI-f) by logistic regression

Explanatory variables	OR (95% CI)	VIF	*p* value
Age (youth vs. elderly (aged 65 or older))	1.088 (0.803–1.474)	1.226	0.585
Gender (male vs. female)	1.800 (1.343–2.412)	1.052	< 0.001
Marital status (unmarried vs. married, divorced, and widowed)	3.050 (2.271–4.097)	1.227	< 0.001
Cause of death (hepatic disease vs. other diseases)	2.475 (1.547–3.960)	1.014	< 0.001

All the reference groups were the latter

*PMI-f* postmortem interval until finding, *OR* odds ratio, *CI* confidence interval, *VIF* variance inflation factors

## Discussion

There have been many studies that have researched solitary deaths [10–18]; however, to our knowledge, this study is the first in Japan to compare the source data of death certificate notifications from the vital statistics survey with emergency transportation and to use these data in a complementary way for analysis. Moreover, one main feature of this study is that, in addition to the more common factors like age and gender, it examined marital status and cause of death, both of which had not been focused upon as much in the previous studies.

Furthermore, we attempted to understand the actual status of solitary deaths by classifying the cases in detail. As described above, we estimated the length of time from the occurrence of death to the discovery of death based on the length of time between the time of death and that of emergency transportation request. We subsequently classified “suspected unnatural death” cases into two groups according to this length of time.

The above analyses indicate that we successfully obtained a new finding regarding the risk factors for solitary death. Previous studies have reported that elderly persons living alone are at a high risk for solitary death [3]. In fact, we found that 65.9% of the cases in the solitary death group were elderly persons aged 65 years or older. In particular, the results from the Pearson’s *χ*^2^ test demonstrate that the time from the occurrence of death to the discovery of death tended to be longer in unmarried, non-elderly males and hepatic diseases. In addition, the logistic regression analysis also showed that an increased likelihood of a prolonged PMI-f was associated with unmarried males with bad hepatic conditions. Various factors might help explain this finding. For example, according to comments in the emergency transportation records, many victims who were discovered relatively shortly after their deaths were found during periodic visits by their family members or relatives, etc. In other words, although it is true that being an elderly person living alone is certainly a risk factor for solitary death, this risk largely depends on the presence or absence of support from surrounding people who live outside the home.

Currently in Japan, the proportion of unmarried persons has been increasing annually for various reasons, including lifestyle diversification and expansion of economic disparity. Unmarried persons, when compared to their married counterparts, have a lower chance of receiving support from families and neighbors, especially when they reach middle- to elderly age. In addition, this social isolation might lead to alcohol abuse, which ultimately leads to hepatic diseases, according to the findings of previous research which stated that individual and community social isolation were associated with increased odds of drinking problems [19, 20]. This is partly because the Japanese public, in general, is culturally more tolerant to alcohol consumption than other substance abuse or smoking, etc. According to a previous research [21], more than 75% of Japanese adolescents have consumed alcohol in their first year of junior high school (when most students are aged 12 years). In the future, welfare administrative authorities should generate preventive measures against solitary death because it is possible that persons of any age living alone, especially unmarried males with hepatic diseases, can be at high risk for solitary death.

Despite the new findings described above, this study faced several limitations. Regarding the solitary death data used in this study, we collected data for only the cases that we were able to link for both the ambulatory care information system and the death certificate notifications. We could not match the remaining cases because the age or gender of the individuals in those cases was unidentified due to advanced decomposition or skeletonization of their bodies. However, there are a considerable number of solitary death cases in the data that we were unable to link. Future studies should aim to obtain the data of these latter cases to understand a more detailed status of solitary deaths. Also, because this study targeted only Yokohama City, it is uncertain whether the conclusions apply to other Japanese regions. In the future, researchers should conduct surveys that are similar to this study in other regions in order to test the validity of our conclusions. Finally, it is worth noting that the data from the ambulatory care information system, as well as the death certificate notifications, were anonymously compared. In other words, the common values between both datasets for the four items (i.e., date of onset, site of onset, age, and gender) were coupled based on pure speculation. In this study, because the quantity of data was relatively small, all data could confidently be matched one-to-one. However, for much larger sample sizes, it might be impossible to anonymously compare the datasets. To further advance studies on this issue, if necessary precautions are taken to protect personal information, breaking anonymity should be considered.

## Conclusion

Unmarried, male sex, and liver diseases are independent risks for solitary death in Yokohama City.

## Abbreviations

CI: Confidence interval
OR: Odds ratio
PMI-f: Postmortem interval until finding
VIF: Variance inflation factors
